# Clinical and ultrasonographic investigations of 30 water buffaloes (*Bubalus bubalis*) with hepatomegaly

**DOI:** 10.14202/vetworld.2019.789-795

**Published:** 2019-06-12

**Authors:** A. M. Abdelaal, M. Abd El Raouf, M. A. Aref, A. A. Moselhy

**Affiliations:** 1 Department of Animal Medicine, Faculty of Veterinary Medicine, Zagazig University, Zagazig, 44519, Egypt; 2 Department of Surgery, Anesthesiology and Radiology, Faculty of Veterinary Medicine, Zagazig University, Zagazig, 44519, Egypt; 3 Department of Anatomy and Embryology, Faculty of Veterinary Medicine, Zagazig University, Zagazig, 44519, Egypt

**Keywords:** buffaloes, fatty liver, hepatic congestion, hepatomegaly, ultrasonography

## Abstract

**Background and Aim:**

Knowledge of normal ultrasonographic dimensions of the liver and associated vascular structures is an important indicator for the diagnosis of hepatic diseases. Enlargement of the liver beyond its normal dimensions is the term of hepatomegaly and ultrasonography is the primary and the suitable diagnostic technique for this condition. Therefore, this study aimed to describe the clinical and ultrasonographic findings of liver diseases causing hepatomegaly in 30 buffaloes as well as to provide a range of liver dimensions and its blood vessel measurements in normal and diseased buffaloes.

**Materials and Methods:**

The study population included 30 buffaloes that were admitted to the clinic of the Faculty of Veterinary Medicine – Zagazig University for investigation of clinical signs associated with gastrointestinal diseases such as anorexia, chronic weight loss, and variable degrees of diarrhea or constipation. The animals were subjected to thorough clinical and ultrasonographic investigations. In addition, 10 healthy buffaloes were investigated ultrasonographically and post-slaughtering for comparison of liver dimensions and physical appearance.

**Results:**

Three conditions causing hepatomegaly were identified in this study as multiple focal hepatic lesions, diffuse fatty liver, and hepatic congestion. Clinically, it was difficult to differentiate between each condition while ultrasonography was the ideal tool for diagnosis after comparing with necropsy as a gold standard tool. Hepatomegaly was recorded in all affected animals with a significant decrease in the size of the portal vein (PV) and caudal vena cava (CVC) in animals affected with multiple focal hepatic lesions and fatty liver disease while the size of the PV and CVC was significantly increased in buffaloes with hepatic congestion.

**Conclusion:**

Ultrasonography can aid to accurately identify buffaloes with hepatomegaly and differentiate between different lesions involved.

## Introduction

Liver has many physiological functions such as metabolism of fats, proteins, carbohydrates, vitamins, bilirubin, and other substances, bile and cholesterol synthesis, blood volume regulation, synthesis of blood coagulation factors, erythrocytes metabolism, removal of blood waste products, and medicaments transport and metabolism [1].

There are many conditions that affect the liver and causing hepatomegaly. On the other hand, not all hepatic lesions lead to hepatomegaly. Hepatomegaly is defined as an enlarged liver beyond its normal dimensions [2]. There are various pathophysiological causes, including high venous pressure, fatty infiltration, and space-occupying lesions within the liver [3,4]. Liver diseases can have great economic importance in bovine industry; however, they have drawn little clinical attention and cannot be noticed due to its specific clinical symptoms [5]. In humans, despite computed tomography and magnetic resonance imaging are the gold standard methods for liver size determination [6,7]. Ultrasonography is the first investigation line for hepatomegaly due to its availability, more rapid, easy, and inexpensive examination procedure [8].

Ultrasonography was used for imaging of the normal structure of the liver and its vessels in cows [9] and ovine [10]. Furthermore, it was used for the diagnosis of several bovine liver diseases [11-18]. Hepatomegaly in buffaloes was diagnosed previously by ultrasound depending only on dilatation of vena cava and portal veins (PV) secondary to the right side heart failure [19]. Based on our knowledge, no previous studies evaluated the hepatic dimensions in buffaloes with different causes of hepatomegaly in buffaloes.

Therefore, the present study was aimed to outline the different causes of hepatomegaly in buffaloes using clinical, ultrasonography, and post-slaughtering findings. In addition, the results of this research comparing the liver’s measurements of normal and diseased buffaloes.

## Materials and Methods

### Ethical approval

This study was approved by and in accordance with the rules of animal use and care ethical committee of the Faculty of Veterinary Medicine, Zagazig University, Egypt.

### Animals

A total of 30 buffaloes of both sexes (26 females and 4 males) with age ranging from 3 to 5 years and 350 to 600 Kg body weight were admitted to the clinic of Faculty of Veterinary Medicine, Zagazig University, with a history of anorexia, chronic loss of body weight, reduced milk yield, abdominal distension, alternative diarrhea with constipation, and failed all attempts of treatment by field veterinarians. As a control group, 10 apparently healthy buffaloes (six males and four females) were examined clinically and ultrasonographically, and liver measurements (weight, length, and width) were performed after slaughtering.

### Clinical examination

All animals were subjected to thorough clinical examinations according to the methods described previously [20]. The clinical parameters, including body temperature, respiratory rate, heart rate, and ruminal movement were recorded.

### Ultrasonographic examination

Ultrasonographic examination of the abdomen and thorax was performed for all animals using a real-time B-mode ultrasound machine (SonoScape A5V, China) with 3.5 convex and microconvex transducers. The liver of animals was examined at the right side from the 12^th^ to 5^th^ intercostal spaces (ICSs). The liver was examined by placing the transducer firmly against the body wall and parallel to the ribs from caudal to cranial and from dorsal to ventral at each ICS starting behind the last rib to the 5^th^ ICS after application of ultrasound coupling gel. A tape measure was used to estimate the distance between the dorsal midline (vertebral column) until the dorsal and ventral margin of the liver at each intercostal, then the size of the liver was assessed by subtracting the dorsal from ventral margin value. The diameters of hepatic blood vessels (caudal vena cava [CVC] and PVs) were determined using the two cursors on ultrasonogram screen [5,9].

### Post-slaughtering examination

All buffaloes under investigations (30 affected and 10 controls) were examined at slaughterhouse to accurately interpret ultrasonographic pictures. Liver weight, length, and width were determined and color, consistency, and lesions were also documented.

### Statistical analysis

The statistical analysis was performed using the SPSS 17.0 software (SPSS, Chicago, IL, USA). The data were expressed as mean ± standard deviation and were analyzed using one-way analysis of variance and LSD *post hoc* tests (p≥0.05).

## Results

Different liver diseases were diagnosed based primarily on ultrasonography and confirmed by post-slaughtering examinations. Multiple focal hepatic lesions, diffuse fatty liver disease, and hepatic congestion were the diagnosed diseases causing hepatomegaly.

### History and clinical investigations

The clinical parameters of the affected animals are illustrated in Table-1. All the affected animals had anorexia, ruminal stasis, and decreased milk yield if the animal was milking. Most animals suffered from chronic weight loss (83.33%). Body systemic reactions were recorded in (6.67%), while icteric mucous membrane was recorded in 10% of the affected animals. Variable degrees of diarrhea and constipation appeared in most affected buffaloes. Other clinical signs, including recurrent tympany, abdominal distension, and dyspnea were also recorded. Fifteen buffaloes had signs of traumatic pericarditis (TP) including jugular engorgement, brisket edema, abduction of the elbow joint, and abnormal heart sounds.

**Table-1:** Clinical investigations of the 30 buffaloes with hepatomegaly.

Items	Hepatic affections			

	Multifocal hepatic lesions	Diffuse fatty liver disease	Hepatic congestion	Total (%)
Anorexia	5	10	15	30 (100)
Systemic reactions*	2	0	0	2 (6.67)
Diarrhea	3	6	5	14 (46.67)
Constipation	2	4	10	16 (53.33)
Recurrent tympany	0	0	11	11 (36.67)
Abdominal distension	2	3	8	13 (43.33)
Weight loss	3	9	13	25 (83.33)
Ruminal stasis	5	10	15	30 (100)
Icteric mucus membrane	2	1	0	3 (10)
Elbow abduction	0	0	15	15 (50)
Brisket edema	0	0	15	15 (50)
Jugular engorgement	0	0	15	15 (50)
Muffled heart sounds	0	0	15	15 (50)
Dyspnea	0	0	6	6 (20)
Total number of animals	5	10	15	30 (100)
Percentages (%)	16.67	33.33	50	100

* Systemic reactions include an elevation of body temperature, pulse rate, and respiratory rate above the reference range of control group (38.239.1°C, 60-82/min, and 22-35/min, respectively)

### Ultrasonographic findings

The ultrasonographic measurements of the normal and the affected animals are summarized in Table-2. Three liver affections accompanied with hepatomegaly were diagnosed ultrasonographically in the affected animals including multiple focal hepatic lesions (16.67%), diffuse fatty liver disease (33.33%), and hepatic congestion (50%).

**Table-2:** Ultrasonographic findings of the normal and affected buffaloes.

Items	Location (ICS)	Normal liver n=10	Multifocal hepatic lesions n=5	Diffuse fatty liver n=10	Hepatic congestion n=15
Liver dimensions	12^th^	22.56±3.05	30.22±3.42	38.33±3.28	43.44±3.56
	11^th^	29.89±1.54	37.33±4.80	39.89±3.41	45.78±3.19
	10^th^	34.11±2.15	40.22±3.77	41.67±4	46.44±2.51
	9^th^	21.44±1.81	29.56±3.84	33.33±2.65	42.11±3.52
	8^th^	17.00±1.87	20.78±2.05	33.56±3.21	38.44±3.50
	7^th^	14.56±2.13	19.89±2.5	26.78±3.03	32.0±2.50
PV	12^th^	3.87±0.30	3.38±0.43	1.03±0.29	5.13±0.63
	11^th^	3.63±0.39	3.26±0.62*	0.76±0.17	4.84±0.88
	10^th^	3.18±0.27	2.41±0.36	0.64±0.23	4.78±0.56
	9^th^	2.62±0.45	1.74±0.48	0.38±0.24	4.47±0.67
	8^th^	1.88±0.27	1.34±0.67*	0.13±0.22	3.59±0.96
CVC	12^th^	3.43±0.19	2.91±0.40	2.04±0.82	4.71±0.42
	11^th^	3.57±0.22	3.18±0.37	1.96±0.49	4.81±0.43
	10^th^	1.62±1.93	0.92±1.41*	Invisible	4.40±0.46
Angle of liver edge		Acute	Slightly acute	Rounded	Rounded
Ascites	Number of animals	0	1	4	13
	Percentage	0	3.33%	13.33%	43.33%
Pleural effusion	Number of animals	0	0	0	6
Pericardial effusion	Number of animals	0	0	0	15

* Non-significant to normal group *P*≥0.05. PV=Portal vein, CVC=Caudal vena cava

### Clinically normal animals (n=10)

**Figure-1:**
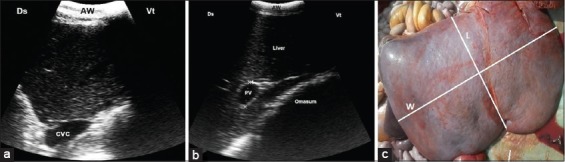
(a) Ultrasonography (US) of the normal liver of buffaloes at the level of the right 11^th^ ICS shows a triangular appearance of caudal vena cava. (b) US of normal portal vein appears with echogenic wall at the level of the 9^th^ ICS. (c) Necropsy of normal liver showing normal brownish color with 25 cm length (L) and 50 cm width (W). AW=Abdominal wall, Ds=Dorsal, Vt=Ventral.

Ultrasonography of the normal animals revealed homogenous echogenic appearance of the hepatic parenchyma over the entire liver with acute angle of its edge. The right kidney was imaged in the hepatorenal impression in the dorsal 12^th^ ICS, while in the 11^th^ and 12^th^ ICS; loops of intestine were visualized adjacent to the liver. From the 8^th^ to 10^th^ ICS, the omasal wall appeared as a thick echogenic line adjacently to the liver, while in the 5^th^ and 6^th^ ICS, the liver parenchyma could not be imaged. The liver size was largest at the level of the 10^th^ to 12^th^ ICS and then decreased cranially with superimposition of the lungs. The CVC was detected in the 12^th^ and 11^th^ ICS in all animals but rarely in the 10^th^ ICS (n=4) and could not be visualized more cranially because it was masked by the lungs. It was situated more dorsal and medial to the PV. It was triangular shaped in cross-section (Figure-1a). Its size measured 3.57±0.22 cm in the 11^th^ ICS. The PV was visualized in the liver with circular echogenic wall in cross-section. The PV was detected from the 12^th^ to 8^th^ ICS. Its size measured 3.87±0.30 cm at the level of the 12^th^ ICS then decreased cranially to be 1.88±0.27 cm at the level of the 8^th^ ICS (Figure-1b). The gallbladder appeared as pear-shaped anechoic fluid-filled vesicle with a thin echogenic wall on the visceral surface of the liver at the 11^th^ to 9^th^ ICS. The intrahepatic and common bile ducts could not be visualized.

### Multiple focal hepatic lesions (n=5)

**Figure-2:**
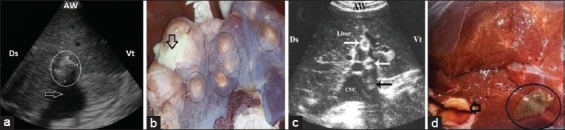
(a) Ultrasonography (US) of liver at the right 8^th^ ICS with a three microconvex transducer shows hyperechogenic structure (circle) with distal acoustic shadowing (arrow). This represents a calcified abscess. (b) Necropsy of a piece of liver shows multiple abscesses (circles) exit pus in cross section (arrow). (c) US of the liver at the level of the right 9^th^ ICS with a convex transducer shows multifocal echogenic patches with ring appearance of bile ducts (white arrow) and distal acoustic shadowing (black arrow). (d) Necropsy of a piece of liver shows adult *Fasciola* (circle) with calcified bile duct (arrow). AW=Abdominal wall, Ds=Dorsal, Vt=Ventral.

Intrahepatic lesions appeared ultrasonographically as multiple echogenic foci of irregular shapes within the hepatic parenchyma with distal acoustic shadowing (Figure-2a and c). Calcifications of the bile ducts appeared in two cases as hyperechogenic ring like in cross-section or tube like in longitudinal section structures with distal acoustic shadowing (Figure-2c). The liver size was significantly increased with accumulation of these foci on liver tissue, and the liver edge appeared with slightly acute angle. The diameter of hepatic vessels was significantly decreased when compared with control group. Ascitic fluid was observed in one animal.

### Diffuse fatty liver disease (n=10)

**Figure-3:**
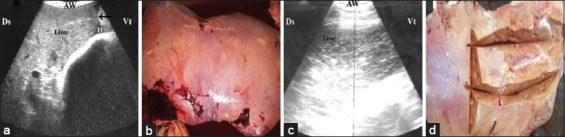
(a) Ultrasonography (US) of a liver at the level of the right 8^th^ ICS shows a mild degree of fatty liver, note the narrow veins, rounded edge (arrow), and scanty amount of anechoic fluid (Fl). (b) Necropsy of a diffuse mild fatty lesion with brownish-yellow color. (c) US of a liver at the levels of the right 10^th^ ICS shows severe fatty infiltration. The hepatic parenchyma appears more echogenic with invisible veins. (d) Necropsy of severe diffuse fatty liver which appears yellowish in color and friable in consistency. AW=Abdominal wall, Ds=Dorsal, Vt=Ventral.

The hepatic parenchyma of the animals suffered from diffuse fatty liver disease appeared ultrasonographically more echogenic than the normal parenchyma and could hardly be distinguished from the adjacent structures in severe cases (Figure-3a and c). The hepatic blood vessels were poorly imaged and the liver angle lost its acute character and curved to become rounded with enlargement of the liver size. In four animals, fatty liver disease was accompanied by anechoic ascitic exudate in the abdomen (Figure-3a).

### Hepatic congestion (n=15)

**Figure-4:**
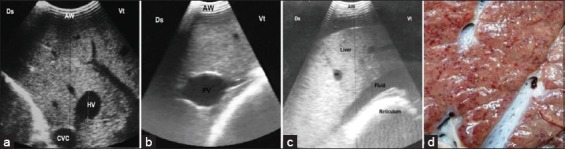
(a) Ultrasonography (US) of a liver with passive congestion at the right 11^th^ ICS, the liver appeared more echogenic with round-shaped caudal vena cava. (b) US of a liver at the 10^th^ ICS appears with dilated portal veins due to passive congestion. (c) US of a liver with passive congestion at the 7^th^ ICS, the liver appeared floated in anechoic ascitic fluid. (d) Necropsy of a piece of liver with chronic hepatic congestion with nutmeg appearance, note the dark red congested regions (accumulation of red blood cells) in hepatic tissues. AW=Abdominal wall, Ds=Dorsal, Vt=Ventral.

**Figure-5:**
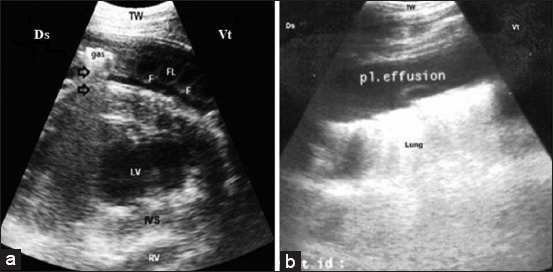
(a) Ultrasonography (US) of a buffalo with pericarditis, the echogenic fibrin interspersed with anechoic exudate, and echogenic gases with dirty shadowing (arrow) in the pericardial sac at the left 4th ICS. (b) US of a buffalo's lung showed clear anechoic fluid in pleural sac at the right 5th ICS. TW=Thoracic wall, Ds=Dorsal, Vt=Ventral, Fl=Fluid, F=Fibrin, LV=Left ventricle, IVS=Interventricular septum, RV=Right ventricle.

Hepatic congestion was seen in buffaloes affected by congestive heart failure due to TP. In these animals, the reticular wall was thickened and corrugated with a reduction in reticular motility (number and strength). Anechoic to hyperechogenic exudate was detected in the abdomen of 13 buffaloes. The liver size was significantly increased with a rounded angle. The diameter of the CVC was significantly increased with a round-shaped appearance in cross-section (Figure-4a). The CVC and PV measurements were significantly increased when compared to the control group (Figure-4a and b). In addition, anechoic fluid was seen around the liver (Figure-4c) and in the abdominal cavity (n=13). Pericarditis was visualized in all cases of hepatic congestion while pleural effusions were observed in six animals (Figure-5).

### Post-slaughtering findings

The post-slaughtering findings of both control and diseased buffaloes are stated in Table-3. Significant increases in weight, length, and width of liver of affected cattle in comparisons with control (Figure-1c) were documented in the current study. The multifocal lesions (n=5) were described as multiple abscesses with caseation (Figure-2b). In two cases with multiple abscesses, the adult worms of fascioliasis were observed in calcified bile ducts (Figure-2d). Fatty liver (n=10) appeared as brownish-yellow to yellowish coloration of the liver with friable consistency (Figure-3b and d), while in case of hepatic congestion (n=15), the liver was observed with dark red congested regions (accumulation of red blood cells) that had a nutmeg appearance (Figure-4d).

**Table-3:** Necropsy findings of 30 buffaloes with hepatomegaly in comparison with 10 controls.

Criteria	Control (n=10)	Buffaloes with hepatomegaly (n=30)		

		Multifocal lesions (n=5)	Diffuse fatty liver (n=10)	Hepatic congestion (n=15)
Liver weight/kg	8.05±1.64	17.4±5.13	18±4.08	18.8±3.59
Liver length/cm	26.8±5.05	39.6±3.36	39.7±1.95	43.07±3.75
Liver width/cm	50.7±7.44	63±5.7	66.8±3.77	70.47±3.11
Characters	Homogenous reddish-brown color with hard consistency.	Multiple abscesses are presented within hepatic parenchyma. Adult *Fasciola* with calcified bile duct is observed in two cases. Liver is hard in consistency	Brownish-yellow to yellowish in color. Friable in consistency	Brownish-yellow with characteristic nutmeg appearance of the liver. Friable in consistency.

## Discussion

In the present study, hepatomegaly was diagnosed in 30 buffaloes suffering from liver diseases. Multiple focal hepatic lesions, diffuse fatty liver disease, and hepatic congestion were recorded in the affected animals. Hepatomegaly accompanying these liver diseases was previously described in humans [3,4].

Anorexia, decrease in milk production, ruminal stasis, and chronic weight loss (83.33%) were the most common clinical signs recorded in the affected buffaloes. These are general clinical signs of most abdominal disorders in bovine and non-specific for liver disease [5]. Only 10% of the affected animals showed signs of icteric mucous membrane that indicated severe liver affection. Diarrhea and constipation were also reported in the affected animals. Abnormal heart sounds, jugular vein engorgement, brisket edema, elbow abduction, dyspnea, and recurrent tympany are characteristic signs for cardiac involvement [21,22] and were found in all animals with TP. Systemic reactions were reported in only two of the affected animals that revealed the ability of buffaloes to overcome pain and their systemic response is different from cows as reported previously [19,23].

Ultrasonography is a reliable, useful, accurate, non-invasive, and easily applicable aid for the diagnosis of hepatic diseases [5,11,15] as well as other bovine thoracic, abdominal, and reproductive disorders [23-27]. The role and accuracy of ultrasonography in the diagnosis of hepatomegaly was investigated in humans [3,4,8,28]. To the best of our knowledge, the present work was the first for studying this condition in buffaloes in details. The liver of the normal buffaloes was visualized in standing position between the 12^th^ and 7^th^ ICS. In cattle, the liver could extend more cranially until the 5^th^ ICS as reported previously [5,9]. In the current study, the largest size of the liver was detected at the 10^th^ ICS, while the largest diameters of PVs were detected at the 12^th^ and 11^th^ ICS and decreased in diameter cranially. The CVC was triangular with the largest diameter at the 11^th^ ICS and could not be seen at the 9^th^-7^th^ ICS. These results prove that the diameter and locations of hepatic blood vessels were nearly similar to those determined previously in cows [5].

As reported in Table-2, there were significant differences between the size of liver in normal and diseased buffaloes. PVs and CVC showed a significant reduction in diameter in buffaloes with multifocal lesion and with diffuse fatty liver while those affected with hepatic congestion showed significant increases in diameters with rounded appearance of CVC. This result is beneficial in differentiation. The narrowing of blood vessels attributed to the space-occupying lesion either abscess, fibrin, or fat leads to compression on blood vessels [5,12,15], while an increase in diameter occurred due to passive congestion of hepatic veins secondary to the right side congestive heart failure [12].

Multiple focal hepatic lesions were identified in five animals. These lesions appeared ultrasonographically as multiple hyperechogenic foci in hepatic parenchyma with acoustic shadowing. Acoustic shadowing under the hyperechogenic foci represented focal calcifications as reported previously [5]. Calcification of the bile duct appeared in cross section as a ring-like echogenic structure with underlying acoustic shadowing. The most common cause of this condition is calcified abscesses or chronic fascioliasis or both. Hepatic abscessation may be due to pyogenic infection of the portal circulation from suppurative lesions anywhere in the body or due to rumenitis arising from lactic acidosis accompanying grain engorgement [29]. *Fusobacterium necrophorum* and *Trueperella pyogenes* are the most common isolated microorganisms from hepatic abscessation in feedlot cattle [30-32].

Diffuse fatty liver disease was recorded in 10 animals, and the hepatic parenchyma appeared ultrasonographically more echogenic than normal, and in severe cases, it could not be identified from neighboring structures. The hepatic angle became round with hepatomegaly. These findings were confirmed at necropsy of the affected animals. Fatty liver is a progressive health problem affecting high lactating animals in early lactation period [33]. The most common risk factors for this condition are negative energy balance, lower concentration of dry matter intake, parturition, stress, and hormonal disturbance [12,34]. It is associated with a high blood concentration of non-esterified fatty acids and beta-hydroxybutyrate that leads to decreasing physiological functions due to their metabolic effects [12].

Hepatic congestion in the current study was secondary to TP that was accompanied by congestion of the systemic circulation and passive congestion of the liver and pleural effusion [13]. Ascites was reported in 18 animals with hepatomegaly that might be attributed to degenerative condition of the liver with improper function, which leads to lowering in intravascular osmotic pressure [5,13,35].

Necropsy findings provided a reference range of control group as stated in Table-3, the liver weight of healthy mature buffaloes was 8.05±1.64 kg; this is nearly similar to those of mature cattle [36]. Moreover, the necropsy findings of both control and diseased buffaloes were the gold standard tool in the current study for evaluation of the results obtained by ultrasonography.

## Conclusion

Clinical investigation is not a diagnostic tool for hepatomegaly in buffaloes, but ultrasonography is a reliable diagnostic tool not only for the assessment of liver lesions but also for evaluation of the liver size. This study provided information about normal ultrasonographic measurements of the liver in buffaloes, which can be used as a guide when investigating buffaloes with suspected liver diseases.

## Authors’ Contributions

All authors designed, planned, drafted, revised, and approved the manuscript. AMA and MA shared in clinical and ultrasonographic examinations. MAA and AAM performed post-slaughtering examinations. All authors read and approved the final manuscript.
